# Low-intensity focused ultrasound attenuates early traumatic brain injury by OX-A/NF-κB/NLRP3 signaling pathway

**DOI:** 10.18632/aging.204290

**Published:** 2022-09-16

**Authors:** Lianghua Huang, Junwei Kang, Gengfa Chen, Wen Ye, Xiangqiang Meng, Qing Du, Zhen Feng

**Affiliations:** 1 Department of Rehabilitation Medicine, First Affiliated Hospital of Nanchang University, Nanchang 330006, Jiangxi, P.R. China

**Keywords:** low-intensity focused ultrasound, traumatic brain injury, orexin-A/NF-κB/NLRP3 pathway

## Abstract

Background: Traumatic brain injury (TBI) is a serious hazard to human health and is characterized by high rates of disability and mortality. It is necessary to explore new effective treatment methods to reduce the impact of TBI on individuals and society. As an emerging neuromodulation technique, ultrasound is used to treat some neurological diseases, but the neuroprotective mechanism of low-intensity focused ultrasound (LIFUS) in TBI remains unclear. We aimed to investigate the protective effects and potential mechanisms of LIFUS in TBI.

Methods: A rat model of TBI was established using the free-fall method. After establishing the TBI model, the hypothalamus region was covered with LIFUS radiation, and an orexin receptor 1 (OXR1) antagonist (SB334867) was injected intraperitoneally. Neurobehavioral examination, Nissl staining, hematoxylin and eosin staining of the brain tissue, and brain water content, were performed 3 days later. Western blotting, quantitative real-time polymerase chain reaction, immunofluorescence staining, and immunohistochemical staining, were used to evaluate the neuroprotective mechanisms of LIFUS.

Results: LIFUS improved tissue damage, neurological deficits, and brain edema. LIFUS can increase the expression of orexin-A (OX-A) and OXR1, significantly inhibit the activation of nuclear factor-κB (NF-κB) protein and nucleotide-binding domain-like receptor protein 3 (NLRP3) inflammasome after TBI, and reduce the release of pro-inflammatory factors after TBI; however, SB334867 can reverse this effect.

Conclusions: This study suggests that LIFUS may play a neuroprotective role by promoting the release of OX-A from the hypothalamus and inhibiting the inflammatory response after TBI through the OX-A /NF-κB/NLRP3 pathway.

## INTRODUCTION

Traumatic brain injury (TBI) remains a major global public health problem [1]. Brain injury often results in various physical and mental sequelae, such as epilepsy, chronic encephalopathy, and depression [2], which cause great harm to sufferers and have a tremendous economic impact on families and the social health system. Therefore, there is an urgent need to explore additional treatment options for TBI. TBI is a complex and heterogeneous mechanical biological condition, and its pathological mechanism is not completely clear. The key to recovery after TBI is reducing toxic substances in the intercellular matrix, eliminating excessive water, inhibiting oxidative stress and inflammation, protecting blood-brain barrier components, and promoting nerve regeneration [3]. In recent years, with emerging studies on neural regulation technology, we observed that it has a certain effect on various neurological diseases, and its treatment of TBI has also become a possibility.

Low-intensity focused ultrasound (LIFUS) neuromodulation technology is an emerging non-invasive neuromodulation technology that combines the advantages of being safe, non-invasive, highly permeable, excellent targeting, and having accurate spatial resolution (a few millimeters) [4]. LIFUS has been proven to be useful in treating various neurological and psychiatric diseases. Recent studies have shown that LIFUS inhibits social avoidance behavior in mice by reducing activation of the microglial inflammatory response, increasing neuronal excitation, and protecting the integrity of the neuronal structure [5]. LIFUS stimulation can also improve memory and cognitive functions in vascular encephalopathy rats [6]. In addition to its mechanical and cavitation effects, LIFUS stimulation can regulate the secretion and absorption of neurochemicals, which is one of the mechanisms of neuroregulation and disease treatment [7]. Previous reports have also confirmed that LIFUS stimulation can reduce neuronal injury and apoptosis in TBI mice and improve their behavioral outcomes by promoting the secretion of brain-derived neurotrophic factor (BDNF) and vascular endothelial growth factor (VEGF) [8, 9]. Using this mechanism of LIFUS and finding an appropriate stimulation target may provide a new approach for TBI treatment.

Inhibition of oxidative stress after TBI is an important method to treat TBI [3, 10]. Studies have shown that oxidative stress after TBI can activate the nuclear factor-κB (NF-κB) to promote the release of inflammatory factors and aggravate inflammatory responses [11]. The NLRP3 inflammasome, a nucleotide-binding domain-like receptor and a downstream transmitter of NF-κB, can be activated by NF-κB to promote the secretion of inflammatory factors [12, 13]. Our team previously discovered that regulation of the NF-κB/NLRP3 pathway can reduce neuroinflammatory response and improve neural function in rats after TBI and orexin-A (OX-A) can reduce the inflammatory response of lipopolysaccharide-induced neural stem cells by regulating the phosphorylation of NF-κB and MAPK/P38/Erk pathways [14, 15]. Zhang C revealed that OX-A can inhibit NLRP3 activity in vascular endothelial cells activated by high glucose induction [16]. We hypothesize that OX-A may be associated with the NF-κB/NLRP3 pathway.

Orexin-A (OX-A) is an excitatory neuropeptide containing 33 amino acids synthesized by the hypothalamus [17]. It is involved in regulating various physiological processes, such as promoting arousal and regulating food intake [18, 19], and has been reported to inhibit neuronal degeneration and death in different central nervous system diseases [20, 21]. Li et al. recently revealed that OX-A alleviates neuroinflammation and improves neurological symptoms after intracerebral hemorrhage in mice [22]. However, there are still limited studies on whether OX-A can reduce inflammation and protect neural function in TBI.

Our team previously observed that deep brain stimulation (DBS), an invasive neuroregulatory technique, can promote the secretion of OX-A to regulate OX-A levels in the brain through targeted stimulation of the hypothalamus [23]. However, whether targeted stimulation of the hypothalamus by LIFUS promote OX-A content in the brain of TBI rats remains unknown.

In this study, we demonstrated that LIFUS targeted hypothalamus stimulation can reduce neuroinflammatory response in the rat at the early stage of TBI and has a protective effect on nerve function. We conducted several detailed experiments and confirmed that LIFUS may promote OX-A secretion and regulate OX-A /NFKB/NLRP3 pathway to achieve this effect. The present study unveils a new stimulation target of LIFUS and a molecular mechanism for the LIFUS neuroprotective role in TBI.

## MATERIALS AND METHODS

### Animals and experimental groups

Male Sprague–Dawley rats of similar body size (7 weeks of age, 250–300 g) from the Nanchang Institute of Experimental Zoology (Nanchang, China) were used in this study. All rats were housed in the Experimental Animal Center of the Nanchang University, kept in an air-conditioned room with a 12-h day and night cycle, fed a standard diet, and had free access to water. The study protocol was approved by the local Animal Research Council and implemented in accordance with the Guidelines of the China Laboratory Nursing Certification Association and the National Institutes of Health (Bethesda, MD, USA). The rats were randomly divided into four groups: sham operation with dimethyl sulfoxide (DMSO) (sham group, n=30, 268.5±10.7 g), TBI with DMSO and sham LIFUS (TBI group, n=30, 266.4±12.3 g), TBI with DMSO and LIFUS (TBI+LIFUS group, n=30, 271.5±11.7 g), and TBI with SB334867, DMSO, and LIFUS (TBI+LIFUS+SB334867 group, n=12, 267.9±12.8 g).

### TBI model

The TBI rat model in this experiment adopted the classical "free-fall model,” the production method described in our research group’s previous report [24]. Briefly, the rats were anesthetized with ether, and a longitudinal incision was made on the scalp along the midline of the skull after disinfection. The rats were then immobilized on a stereoscopic device using two ear rods and nose forceps. A metal cylinder hammer, weighing 400 g, was then dropped freely from a height of 45 cm along a vertical tube to impact the intersection of the left cerebral hemisphere, 1 mm in front of the coronal suture and 2 mm from the midline. The mice in the sham group were only pierced on the skin. After the operation, the skin was disinfected and sutured again, and mice were placed in a heated cage to maintain body temperature while recovering from anesthesia.

### Intraperitoneal drug injection

The anesthetized rats were injected intraperitoneally. SB334867 (ab120164, Abcam, USA), an orexin receptor 1 (OXR1) selective antagonist, was dissolved in 5% DMSO to the drug solution (10 mg/ml), and then 100 μl was injected 10 min before LIFUS induction in the SB334867 group; the other three groups received the same volume (100 μl) of 5% DMSO [25].

### LIFUS

**Figure 1:**
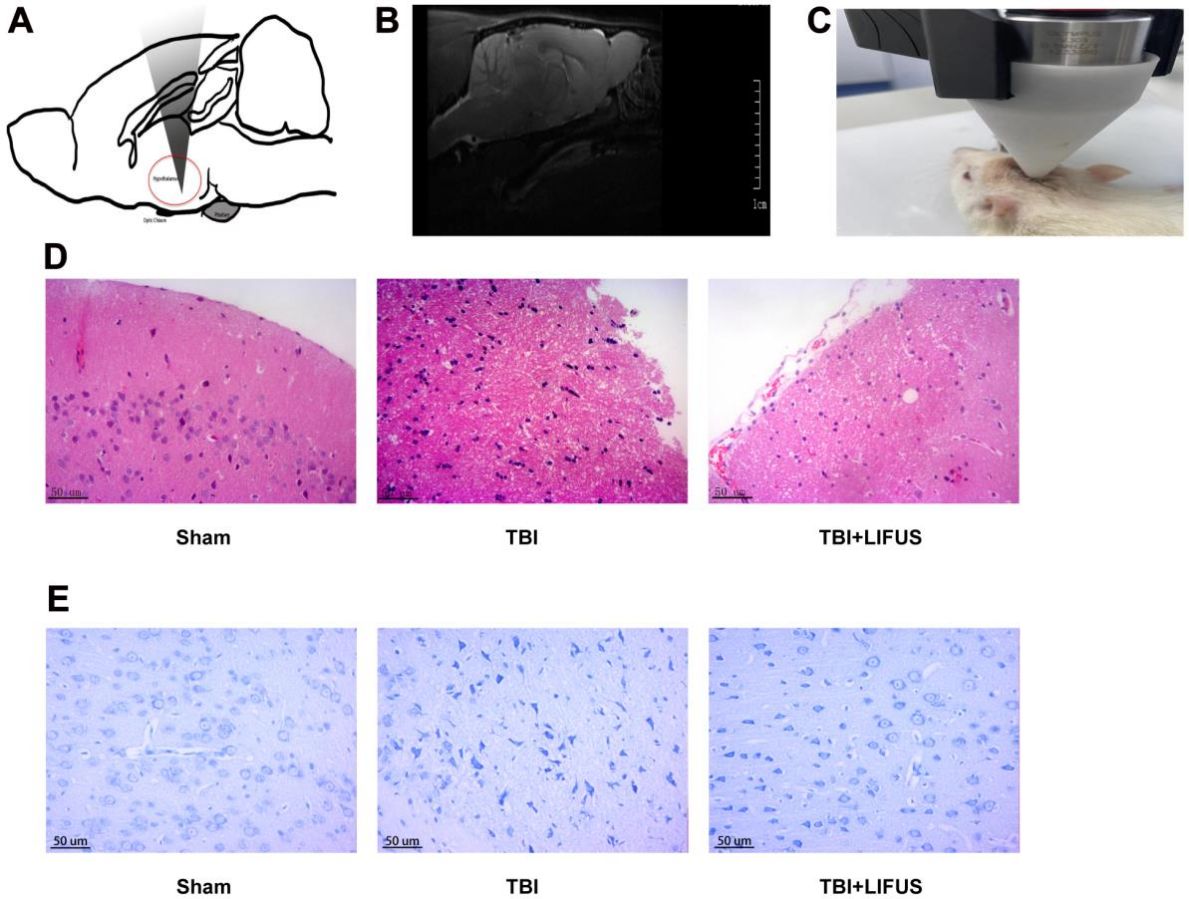
**Low-intensity focused ultrasound (LIFUS) relieves tissue damage, neuronal degeneration and necrosis.** (**A**) Schematic diagram of the relationship between ultrasonic path and rat brain anatomy. The inset shows a vertically incident ultrasonic beam. (**B**) A representative magnetic resonance imaging image of a rat brain. (**C**) LIFUS in rats. (**D**) Representative hematoxylin and eosin staining images of three groups of cortical tissues showing areas of necrosis, Scale=50 um. (**E**) Representative Nissl staining of three groups of cortical tissues demonstrates necrotic degeneration of neurons, Scale=50 um.

The ultrasonic pulse signal generated by the ultrasound generator (NTK-UNS-01 system, Jiangxi Brain Regulation, China) was transmitted to the ultrasonic transducer (V301-SU; Olympus, Japan). The transducer measures a focal length of 33 mm and is connected with a replaceable 3D-printed conical collimator. There are three models of vertebra quasi-actuators, with heights of 24 mm, 25 mm, and 26 mm. We used magnetic resonance imaging (MRI) for a small animal system (pharmascan70/16us, Bruker, Germany) to scan the rat brain (Figure 1B) to coordinate the brain stereo position device (ZH-blue star, Anhui Zhenghua, China) and the rat brain three-dimensional anatomical map [26] to identify the hypothalamus. LIFUS was administered 20 min after TBI, the quasi-actuator was filled with an ultrasonic coupling agent, the rat head was held tightly (Figure 1C), and the hypothalamus region was covered by ultrasound by adjusting the distance of the ultrasonic probe focus in the brain with quasi-actuators. The ultrasonic wave frequency was set to 500 Hz (the fundamental frequency penetrated the skull best in the frequency range of 440 to 700 KHz [27]), on-break ratio was 5%, pulse repetition frequency (PRF) was 250 Hz, and total ultrasonic duration was 400 ms. The spatial-peak temporal-average intensity in degassed water was 518 mW/cm^2^ measured using a hydrophone (HNR-1000, Acoustics Lt, Dorchester, United Kingdom). Each rat was stimulated for 30 min in a day for three consecutive days. In sham LIFUS, the ultrasonic probe was only placed on the rat head without starting the ultrasound machine, and no ultrasonic stimulation was produced by the probe.

### Modified neurological severity scale (mNSS)

Three days after TBI, mNSS was detected to evaluate the neurological deficits in rats in each group (n = 6). The mNSS test includes 10 components: motor, sensory, balance, and reflexes. The score ranged from 0 to 18, and the severity of neurological impairment increased. One point is scored for each abnormal behavior or untested reflex, with an overall score of 0 being normal, 1–6 mild, 7–12 moderate, and 13–18 severe. All the neurobehavioral tests were performed by two researchers, who were blinded to the group they were assigned to.

### Balance beam test

The balance beam device was mounted on the table’s surface so that the crossbar was 20 cm away from the table’s surface. A soft cushion was laid under the crossbar to prevent the rats from falling. The four limbs of the rats were placed at the beginning of the crossbar, and the rats were given appropriate sono-optic stimulation to push them forward and into the dark box at the end of the crossbar. The condition in which the hind limbs slide down the crossbar when the rat cannot walk on the crossbar was called a slip. The operator recorded the number of slips of the rats from the beginning to the end of the crossbar and rated the score. With each rat, the experiment was conducted three times, the score was recorded three times, and the average value was calculated [28].

### Quantitative real-time polymerase chain reaction (qRT-PCR)

Three days after treatment, total RNA from pericontusion traumatic brain tissues (each group n = 6) was extracted using TRIzol reagent (Invitrogen). Total RNA was reverse transcribed to synthesize cDNA using a commercial kit (Takara). qRT-PCR was performed using a SYBR Green PCR Master Mix (TransGen Biotech) following the manufacturer’s instructions, and PCR was performed using the CFX96 Real-Time PCR Detection System. The primers used are listed in Table 1. The amplification procedure was as follows: denaturation at 95° C for 15 min, followed by 40 cycles of annealing at 95° C for 10 s, and extension at 60° C for 32 s. The melting curve started at 95° C for 15 s, at 60° C for 1 min, and ended at 95° C for 15 s.

**Table 1:** The primer sequences for PCR amplification.

**Gene target**	**Primer sequence**	**Tm (° C)**
TNF-α	F: 5′-CAGCCAGGAGGGAGAAC-3′	63.9
	R: 5′-GTATGAGAGGGACGGAACC-3′	63.9
IL-1β	F: 5′-CCCTTGACTTGGGCTGT-3′	64.2
	R: 5′-CGAGATGCTGCTGTGAGA-3′	64.2
IL-18	F: 5′-AACGAATCCCAGACCAGAC-3′	64.1
	R: 5′-AGAGGGTAGACATCCTTCCAT-3′	64.5
NLRP3	F: 5′-TGTTGTCAGGATCTCGCA-3′	63.4
	R: 5′-AGTGAAGTAAGGCCGGAAT-3′	63.5
GAPDH	F: 5′-TCTTTGCTTGGGTGGGT-3′	63.7
	R: 5′-TGGGTCTGGCATTGTTCT-3′	63.7

Tm, melting temperature; GAPDH, glyceraldehyde 3-phosphate dehydrogenase; NLRP3, nucleotide-binding domain-like receptor protein 3; IL, interleukin; TNF, tumor necrosis factor.

### Brain water content

The rats were anesthetized with ether; thereafter, the brain tissue (each group n = 6) was cut off quickly. The brain tissue was then divided into left and right hemispheres along the middle line with a scalpel. Subsequently, it was weighed using an electronic balance to obtain the wet weight. The samples were then dried in an oven at 100° C for 24 h and weighed again to determine the dry weight. Finally, the water content of the brain was calculated using the formula for the percentage of water in the brain: brain water content (%) = ([wet weight − dry weight]/wet weight) × 100%.

### Western blot

The rat brain (each group n = 6) was removed, and the damaged and surrounding tissues were selected for protein extraction using a protein extraction kit (KTP 3001, Exkine, China). This procedure was performed according to the manufacturer’s instructions. Protein samples were stored at -80° C. Protein samples were loaded into polyacrylamide gel channels for electrophoresis. After protein separation, the PVDF membrane was covered on the gel for protein transfer and soaked in a skim milk solution (5%) for 1 h after transfer. Subsequently, the primary antibody was diluted with an antibody diluent (AR1017, BOSTER, China) as per manufacturer’s instructions. The PVDF membrane was then placed into it and incubated at 4° C for 12 h. Following incubation, the membranes were washed with TBST three times and then incubated with the secondary antibody with skim milk solution (5%) at room temperature at a concentration of 10000:1 for 1 h. Subsequently, the PVDF membrane was washed with TBST three times, and a chemiluminescence solution (S6009M, UElandy, China) was added to the luminescence instrument to develop and take photos. The ImageJ software was used to calculate the relative density of the grayscale of protein bands. Primary antibodies for western blot were anti-OX-A (1:1000, AB3098, Sigma, USA), anti-orexin receptor 1 (OXR1) (1:1000, 18029R, BIOSS, China), anti-tumor necrosis factor (TNF)- α (1:1000, AF7014, Affinity, USA), anti-interleukin (IL)-18 (1:1000, DF6252, Affinity, USA), anti-IL-1β (1:1000, AF5103, Affinity, USA), anti-NLRP3 (1;500, ab214185, Abcam, USA), anti-NF-κBp65 (1:1000, ab16502, Abcam, USA), anti-LaminB (1;1000, ab16048, Abcam, USA), and anti-β-actin (1:1000, T0022, Affinity, USA). The secondary antibodies used were anti-rabbit (1:10000, ab205718 Abcam, USA) and anti-mice (1:10000, ab205719, Abcam, USA).

### Hematoxylin and eosin staining (H&E staining)

Three days post-injury, the rats were decapitated after induction of anesthesia with ether, and the brain tissues (each group n=6) were harvested. The tissue was immersed in 4% polyformaldehyde at room temperature for 24 h, dehydrated with 70%, 80%, 90%, 95%, and 100% ethanol for 30 min each, soaked in xylene for 10 min, and finally embedded in paraffin and cut into 4-μm-thick slices. Paraffin-embedded brain tissues were stained with H&E. Briefly, the slices were stained with hematoxylin solution for 3 min, stained in eosin solution for 5 min, dehydrated with ethanol again, and sealed with neutral gum. The slides were then examined under a light microscope.

### Nissl staining

Paraffin-embedded tissue sections, after dewaxing and hydration, were stained with Nishil dye (toluidine blue) for 5 min and cleaned with distilled water three times for 20 s each. The cleaned brain slices were treated with xylene for 5 min; thereafter, the brain slices were dehydrated with different concentrations of ethanol and sealed with neutral gum. The slides were then examined under a light microscope.

### Immunofluorescence staining

The protein levels of OXR1 were detected by immunofluorescence staining of paraffin-embedded sections. Sections were cut 5 μm thick, dewaxed in xylene, dehydrated in 100%, 95%, 85%, and 80% ethanol, and immersed in EDTA antigen repair solution for 30 min in a hot water bath. After cooling naturally, the slices were placed in phosphate-buffered saline (PBS; pH 7.4) and washed by shaking on a decolorizing shaker three times for 5 min each. Following drying, after which the bovine serum albumin (BSA; G5001, Servicebio, China) was sealed for 30 min, the sections were incubated with primary antibodies at 4° C. It was washed and decolorized with PBS (PH 7.4) and covered with a secondary antibody (1:300, Gb21303, Servicebio), and incubated at ambient temperature in the dark for 50 min. DAPI (G1012, Servicebio) solution was added after PBS (pH 7.4) decolorization, then incubated at room temperature and away from light for 10 min. Finally, the self-quenching agent was added for 5 min, and the water was rinsed for 10 min. The slides were examined under a light microscope. Primary anti-bodies for Immunofluorescence staining were: anti-OXR1 (1:200, 18029R, BIOSS, China).

### Immunohistochemical staining

OX-A protein levels were detected by immunohistochemical staining of the paraffin-embedded sections. Dewaxing dehydration and antigen repair were the same as the above immunofluorescence steps. Subsequently, the sections were placed in 3% hydrogen peroxide solution and incubated at room temperature for 25 min in the dark to block endogenous peroxidase, then washed with PBS (pH 7.4) and 3% BSA (G5001, Servicebio, China) to cover the tissue evenly. The sections were sealed at room temperature for 30 min, and then placed flat in a wet box with primary antibodies (1:200, 15509R, BIOSS, China) at 4° C and incubated overnight. The incubated sections were rinsed with PBS (PH 7.4) three times, 5 min each. Following drying, a secondary antibody (GB23303, Servicebio) was added, and the sections were incubated for 50 min at room temperature. Finally, DBA (G1211, Servicebio,) and hematoxylin staining were successively added. The slides were observed under a light microscope after sealing.

### Statistical analyses

To detect the distribution pattern of the data, analysis of variance (ANOVA) was used for data that matched the normal distribution, and the Kruskal–Wallis test was used for non-normally distributed data. Statistical analysis was performed using the GraphPad Prism 8.0 software (GraphPad Software, San Diego, California, USA). Statistical results are expressed as mean ± standard deviation (SD), and the results were considered statistically significant when the p-value was less than 0.05.

## RESULTS

### LIFUS reduced tissue damage, necrotic neuronal degeneration, neurological deficits, and brain water content

**Figure 2:**
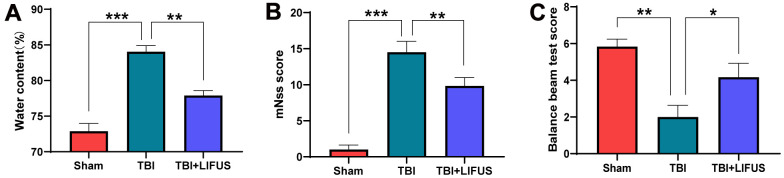
**Low-intensity focused ultrasound (LIFUS) relieves cerebral edema and neurological deficits.** (**A**) The water content of each group was measured 3 days after traumatic brain injury. (**B**, **C**) Three days after traumatic brain injury, neurological function was analyzed using the Modified Neurological Severity Scale (mNSS) and balance beam test. (n=6, *P<0.05, **P<0.01, ***P<0.001).

The TBI model was established, and after neurological function testing, the brain tissue was harvested for histological examination to determine whether LIFUS had a therapeutic effect on TBI. H&E and Nissl Staining results revealed that in the sham operation group, abundant neuron structures were observed in the brain tissues, with complete morphology and structure of all cells, dense intercellular arrangement, and normal cell volume. The TBI group had loose structure, obvious infiltration of inflammatory cells, extensive vacuolar changes, The TBI group had loose structure, obvious infiltration of inflammatory cells, extensive vacuolar changes, and cell necrosis of neurons, and was accompanied by cytoplasmic atrophy, oval or triangular nucleus and typical neurodegeneration. However, treatment with LIFUS significantly improved these conditions (Figure 1D, 1E). By measuring the water content of rat brain tissue, we observed that the brain water content of the TBI group was significantly higher than that of the sham operation group (*p* < 0.001). LIFUS treatment significantly reduced the water content of the brain tissue after TBI and improved cerebral edema (*p* < 0.01) (Figure 2A). The mNSS (Figure 2B) and balance beam tests (Figure 2C) were performed in the rats to evaluate the effects of LIFUS on neurological function after TBI. Compared with the sham operation group, the TBI group had a higher mNSS score (*p* < 0.001) and a lower balance beam score (*p* < 0.01), while the LIFUS group had a significantly lower mNSS score (*p* < 0.01) and a higher balance beam score (*p* < 0.05) compared with the TBI group, suggesting that LIFUS treatment promoted neurological recovery after TBI.

### LIFUS increased the expression of OX-A and OXR1

**Figure 3:**
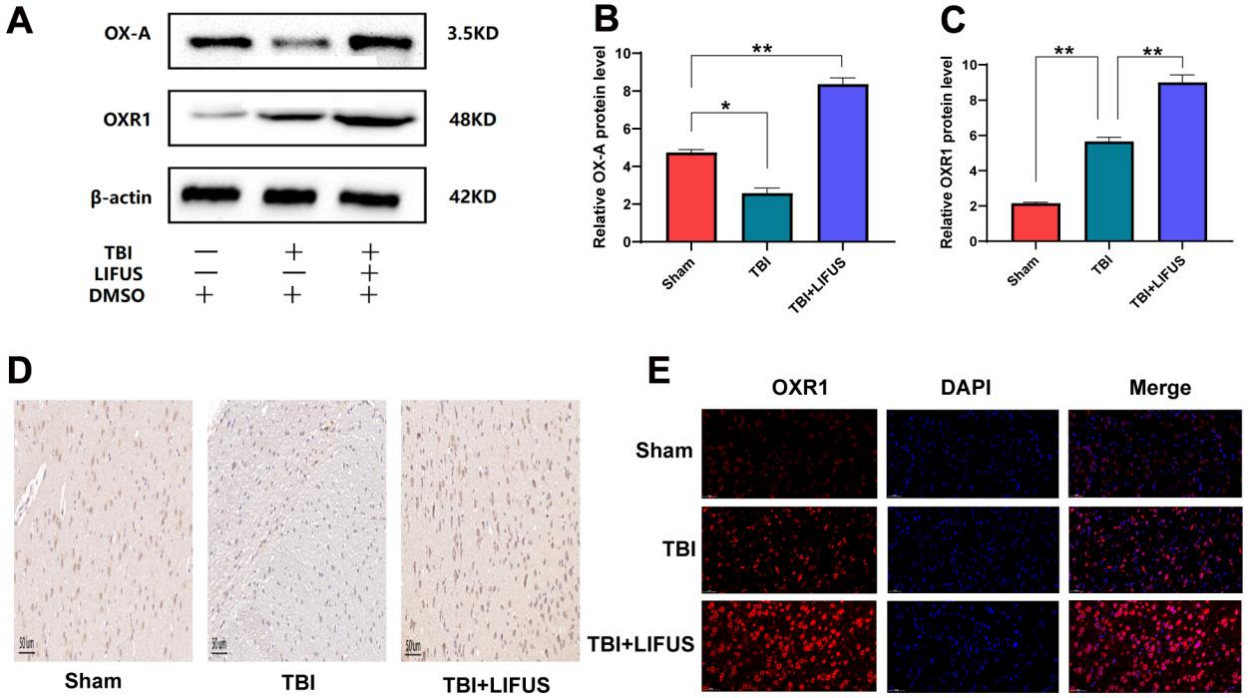
**Low-intensity focused ultrasound (LIFUS) can increase the expression of orexin-A (OX-A) and orexin receptor 1 (OXR1) in traumatic brain injury (TBI) rat brain tissue.** (**A**) OX-A and OXR1 represent western blot protein bands. (**B**, **C**) represents the gray values of the OX-A and OXR1 bands, respectively. (**D**) Representative immunohistochemical staining and relative protein expression of OX-A. (positive expression was brownish yellow. Scale=50 um). (**E**) OXR1 representative immunofluorescence staining. (OXR1 staining is red, and all nuclear DAPI staining is blue. Scale=50 um). Results are expressed as mean±standard deviation (n=6, SD, *P<0.05, **P<0.01, ***P<0.001).

To test whether LIFUS stimulation of the hypothalamus can affect OX-A expression, the content of OX-A in tissues was measured using western blotting and immunohistochemical staining, and the western blot results were quantified. Finally, we observed that the results of western blot and immunohistochemical staining were consistent: western blot results suggested that the OX-A content of the brain tissue in the TBI group was lower than that in the sham group (*p* < 0.05); however, the concentration of OX-A in the TBI+LIFUS group was significantly higher than that in the sham group (*p* < 0.01) (Figure 3A, 3B). Immunohistochemical staining results showed that OX-A was highly expressed in the TBI + LIFUS group, moderately expressed in the sham group, and low in the TBI group (Figure 3D). Meanwhile, we detected changes in OXR1 expression by western blotting and immunofluorescence staining, which suggested that the content of OXR1 in the TBI group was enhanced compared with that in the sham group (*p* < 0.01), and further increased in the TBI+LIFUS stimulation group (*p* < 0.01) (Figure 3A, 3C). Immunofluorescence staining showed that OXR1 was highly expressed in the TBI + LIFUS group, moderately expressed in the TBI group, and low in the sham group (Figure 3E). Based on the above experimental results, it can be concluded that LIFUS treatment promotes the expression of OX-A and OXR1.

### LIFUS inhibited the production of pro-inflammatory cytokines in TBI models

**Figure 4:**
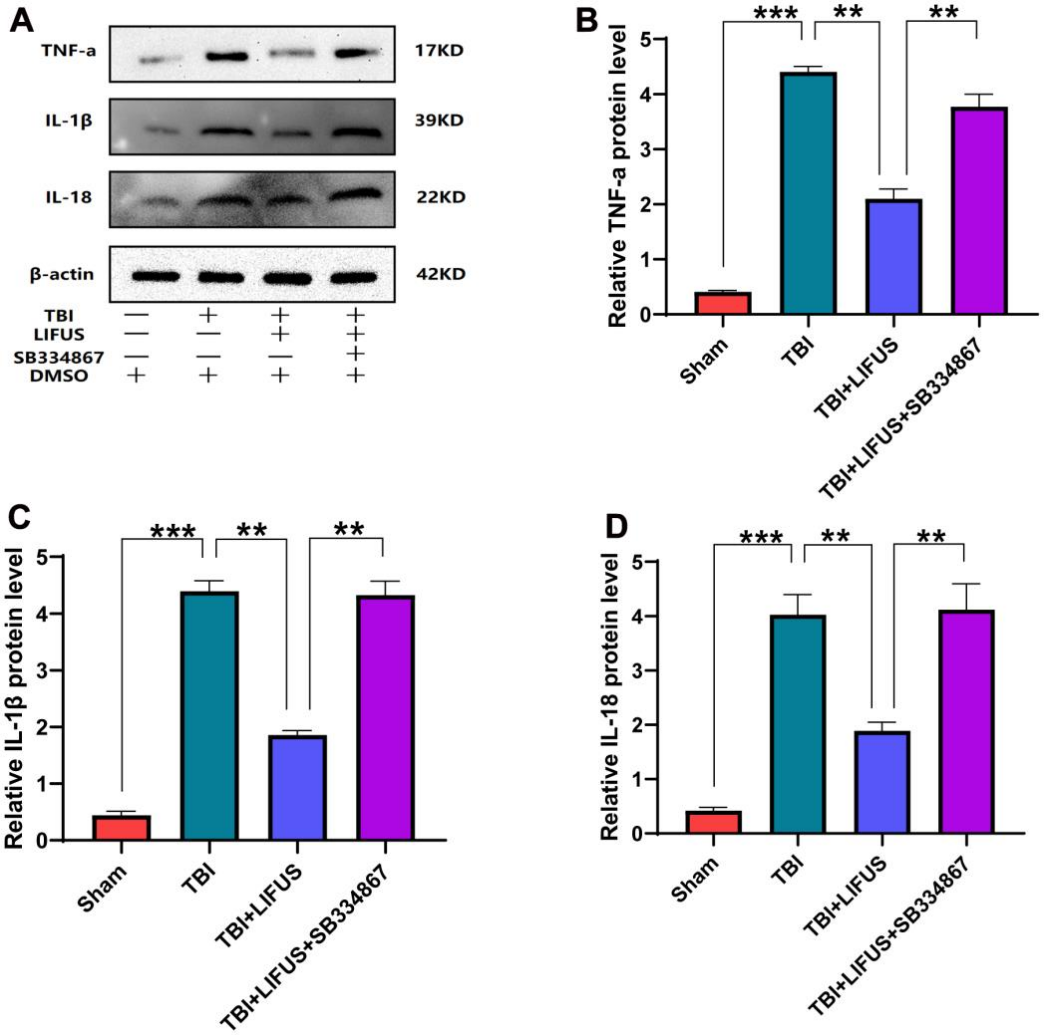
**Low-intensity focused ultrasound (LIFUS) inhibited the levels of pro-inflammatory factors in traumatic brain injury (TBI) rat models by orexin-A (OX-A).** (**A**) tumor necrosis factor-a (TNF-a), interleukin-1β (IL-1β), and interleukin-18 (IL-18) represent western blot protein bands, (**B**–**D**) are the gray values of TNF-a, IL-1β, and IL-18 bands, respectively, Results are expressed as mean±standard deviation (n=6, SD, *P<0.05, **P<0.01, ***P<0.001).

**Figure 5:**
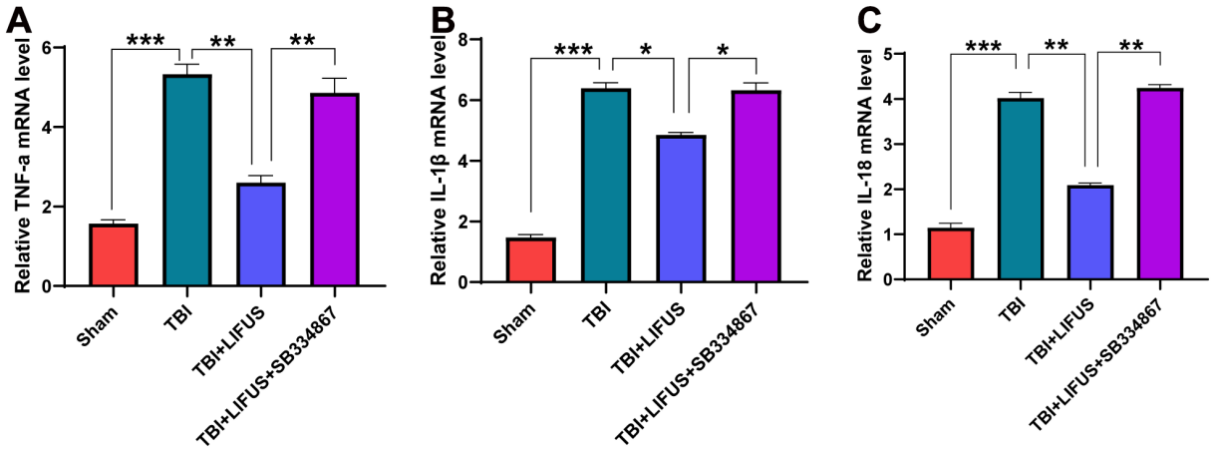
**Relative mRNA levels.** (**A**) tumor necrosis factor-a (TNF-a), (**B**) interleukin-1β (IL-1β), (**C**) interleukin-18 (IL-18). Results are expressed as mean ± standard deviation (n=6, SD; *P < 0.05, **P <0.01, ***P < 0.001).

The inflammatory response after TBI is an important factor in neurological impairment caused by TBI. We detected the effect of LIFUS on pro-inflammatory factors, quantified the western blot results (*p* < 0.05) (Figure 4A–4D), and found that the protein levels of TNF-α, IL-1β, and IL-18 were consistent with the mRNA levels (*p* < 0.05) (Figure 5A–5C). Compared with the sham group, TNF-α, IL-1β, and IL-18 levels were significantly increased in the TBI group. The levels of these pro-inflammatory factors were reduced in the LIFUS group compared to the TBI group, but the effect of LIFUS was reversed when SB334867 was added to block OX-A function, suggesting that the anti-inflammatory effect of LIFUS may be generated through OX-A.

### LIFUS inhibits the activation of NF-κB/NLRP3 inflammasome in TBI models

**Figure 6:**
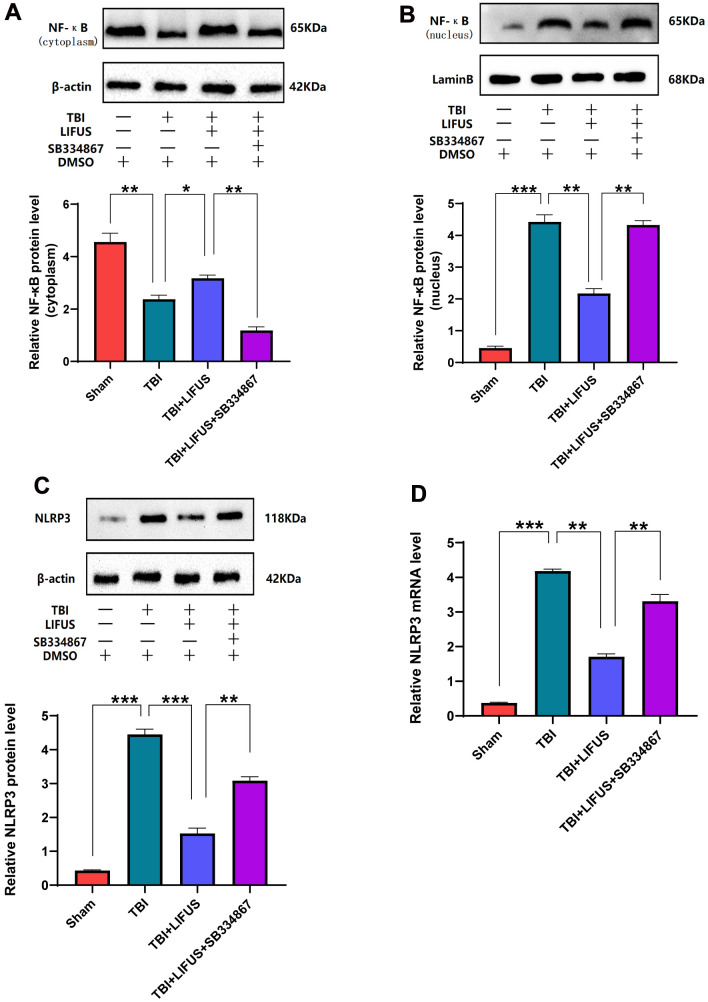
**Low-intensity focused ultrasound (LIFUS) inhibited the activation of NF-κB (nuclear factor κB) and nucleotide-binding domain-like receptor protein 3 (NLRP3) inflammasome after traumatic brain injury (TBI) by orexin-A (OX-A).** (**A**) NF-κB protein levels in the cytoplasm. (**B**) NF-κB protein levels in the nucleus. (**C**) Protein levels of NLRP3. (**D**) NLRP3 corresponding mRNA levels (n=6, SD; *P < 0.05, **P <0.01, ***P < 0.001).

The NF-κB/NLRP3 pathway plays an important role in brain injury after TBI. The content of NF-κB in the cytoplasm and nucleus was measured using western blotting. The results showed that compared with the sham operation group, the protein level of NF-κB in the cytoplasm was significantly decreased, and the protein level in the nucleus was significantly increased under TBI. LIFUS inhibited the transfer of NF-κB from the cytoplasm to the nucleus; however, this effect was reversed after the addition of SB334867 (*p* < 0.05) (Figure 6A, 6B). Western blotting analysis showed that NLRP3 expression and mRNA levels were consistent. The NLRP3 level in the TBI group was higher than that in the sham group, and the NLRP3 level in the LIFUS group was lower than that in the TBI group; however, the NLRP3 level in the TBI+LIFUS+SB334867 group was higher than that in the LIFUS group (*p* < 0.05) (Figure 6C, 6D). The results revealed that LIFUS may inhibit the activation of the NF-KB/NLRP3 inflammatory pathway through OX-A.

## DISCUSSION

**Figure 7:**
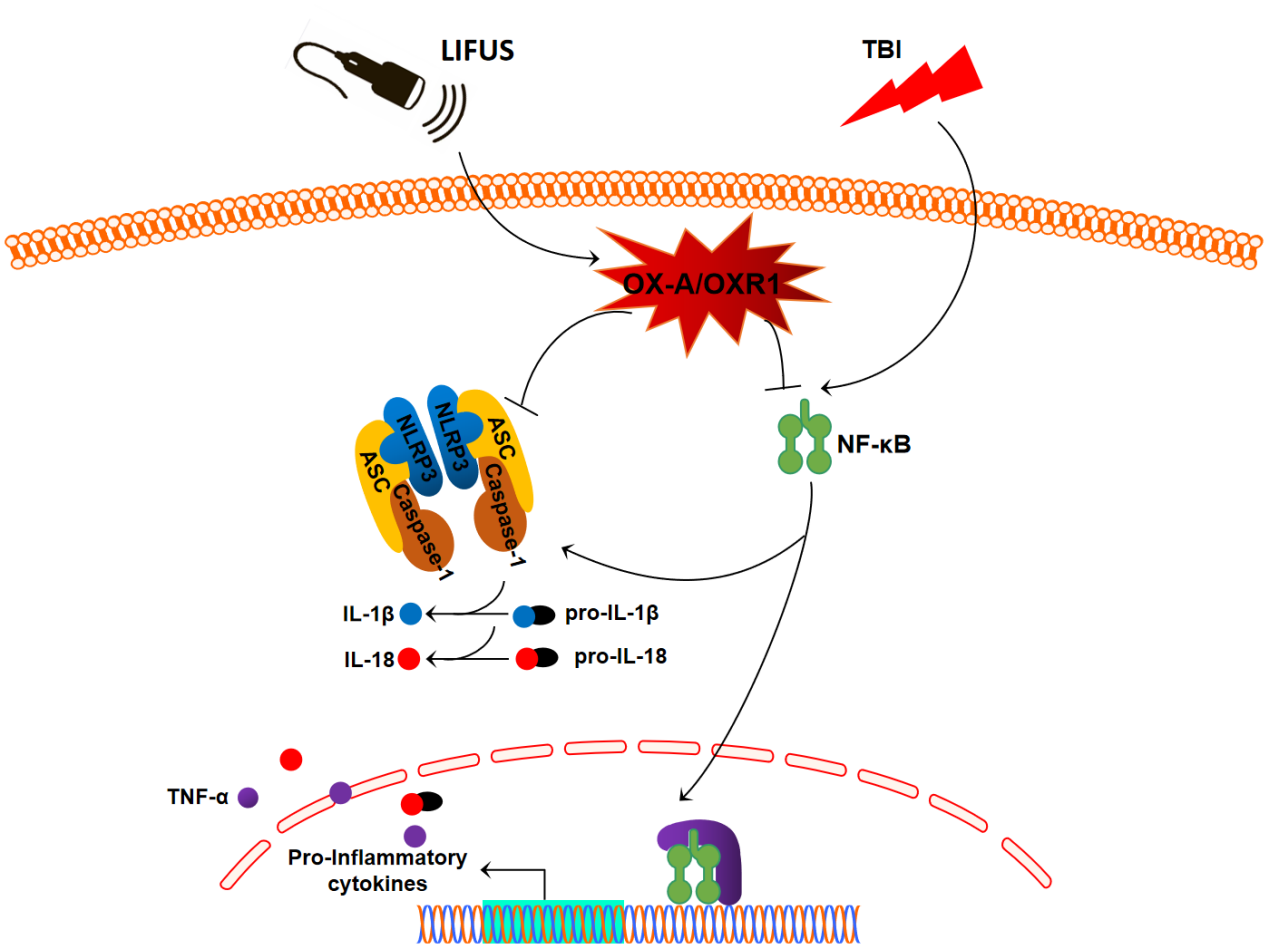
**Schematic diagram depicting the potential mechanism and protective effects of low-intensity focused ultrasound (LIFUS) on traumatic brain injury (TBI).** LIFUS can significantly inhibit inflammatory response after TBI through the OX-A/NF-κB/NLRP3 signaling pathway.

In this study, we observed that the stimulation of the hypothalamus by LIFUS can reduce the content of pro-inflammatory cytokines in damaged tissues of the TBI rat model, alleviate brain injury, and improve neural function. Meanwhile, it was observed that the OX-A content decreased and OXR1 content increased in injured tissues after TBI; however, LIFUS stimulation promoted the secretion of OX-A and further increased the content of OXR1. OX-A is an important pathway in regulating the inflammatory response. In this study, we demonstrated the effectiveness of LIFUS in improving inflammatory response in TBI by regulating the OX-A/NFKB/NLRP3 signaling pathway (Figure 7).

In recent years, with the emergence of ultrasonic focusing and imaging technology, the application value of LIFUS in the field of central nervous regulation has attracted widespread attention. LIFUS is non-invasive, accurate, and safe, and can stimulate or inhibit any of the target areas of the brain. This new technology has many potential uses, including in the treatment of many mental and neurological disorders [7]. At present, the neural regulatory mechanism of LIFUS is still being studied, and some achievements have been made. Previous studies have shown that the mechanical [29, 30] and cavitation [31, 32] effects of ultrasound are the mechanisms by which LIFUS exerts neural regulation. However, with the development of research, it has been observed that the changes in neurochemicals caused by LIFUS are also an important mechanism [7]. It has been reported that LIFUS stimulation of the thalamic region of rats can increase the extracellular concentration of dopamine and 5-hydroxytryptamine and decrease the concentration of gamma-aminobutyric acid (GABA) in the frontal lobe [33]. Existing reports on ultrasound and TBI are related to this mechanism [8, 9]. Based on this mechanism, the hypothalamus of the TBI rat was stimulated in this experiment and it was observed that LIFUS could increase the expression of OX-A.

Recent studies have shown that neuropeptide OX-A is associated with various neurological diseases, such as Alzheimer’s disease [34], Parkinson’s disease [35], and epilepsy [36]. Dohi et al. revealed that the amount of OX-A in the cerebrospinal fluid of patients with subarachnoid and cerebral hemorrhages decreases [37, 38]. Ang BT et al. confirmed that the severity of subarachnoid hemorrhage appears to be negatively correlated with OX-A in the cerebrospinal fluid [39]. These findings suggest that OX-A may play an important role in brain injury. This experiment showed that the amount of OX-A in the TBI model was significantly lower than that in the sham group, which was consistent with the results of a previous study; however, the changes in the neurological function scores and histological results of rats improved after LIFUS treatment suggested that LIFUS had the effect of alleviated TBI injury and neuroprotection, which was accompanied by an increase in the OX-A content detected in the tissues, suggesting that OX-A may play a beneficial role in TBI, which was also consistent with some previous research results. Kitamura et al. revealed that the injection of exogenic OX-A in the brain can reduce the cerebral infarction area and nerve protection [40]. Xiong et al. used OX-A gene-knocked rats and wild-type rats to develop a model of middle cerebral artery occlusion and found that OX-A could play an anti-inflammatory role in reducing cerebral infarction area and neuroprotection by inhibiting myeloperoxidase activity [41].

Mihara et al. found that in a TBI rat model, OXR1 content in the brain tissue gradually increased 6 h after injury, reached a peak on the first day, and then gradually decreased from the second day to the seventh day [42]. Dohi et al. also monitored the changing trend of OX-A and OXR1 content in the cerebrospinal fluid of cardiac-arrest rats and obtained similar results to those of Mihara et al. [43]. In this study, it was also observed that OXR1 increased after TBI, and that LIFUS could further improve the expression of OXR1, which may be a positive feedback effect caused by the increase in OX-A content.

Following TBI, the release of pro-inflammatory factors can aggravate nerve cell injury [3]. For example, IL-1β can trigger the inflammatory cascade induced by TBI, leading to enlargement of the TBI injury area and aggravation of cognitive loss [44]. In this study, we observed that treatment with LIFUS reduced the levels of pro-inflammatory cytokines TNF-α, IL-1β, and IL-18; however, this effect was blocked after the addition of SB334867, suggesting that LIFUS acts as an inflammatory suppressor through OX-A. Some previous reports have also confirmed the anti-inflammatory effects of OX-A. For example, Zhang et al. revealed that OX-A inhibits the activation of MAP kinase P38 and NF-κB, thereby reducing the levels of the pro-inflammatory cytokines TNF-α, IL-6, and IL-1β mediated by low-density lipoprotein and alleviates its inflammatory response to vascular endothelial cells [45].

NF-κB/NLRP3 is a classic signaling pathway involved in many inflammatory diseases [13]. NF-κB can be activated by TBI-induced oxidative stress, and activated NF-κB can promote the transcription of pro-inflammatory factors that play key roles in inflammation-related diseases [46, 47]. Casili et al. found that dimethyl fumarate could inhibit the activation of NF-κB, reduce the levels of TNF-α and IL-1β, and play a role in alleviating nerve tissue injury and neuroprotective function in TBI rats [11]. However, it can only promote the transcription of inflammatory factors when NF-κB is transferred from the cytoplasm to the nucleus. Therefore, the activation of NF-κB can be determined by measuring the cytoplasm and nucleus [14]. In this study, we observed that LIFUS inhibited the activation of NF-κB and reduced the secretion of pro-inflammatory cytokines. However, this effect was reversed after the addition of the SB334867; therefore, LIFUS may achieve this function through OX-A.

The NLRP3 inflammasome is a polyprotein oligomer containing an NLRP3 scaffold, apoptosis-associated speck-like protein adapter (ASC), and procaspase-1, which can be activated by oxidative stress response after TBI to aggravate brain damage caused by TBI and hinder the recovery of neurological function [48]. NLRP3 can also be activated by NF-κB as a downstream mediator of the NF-κB pathway, and activated NLRP3 can promote the release of IL-1β and IL-18 and enhance the inflammatory response [16, 49]. Our results in this experiment are consistent with those of previous studies. We also discovered that LIFUS inhibited NLRP3 inflammasome activation and reduced the release of inflammatory cytokines. However, this effect was reversed by the SB334867; therefore, LIFUS may have inhibited the activation of NLRP3 by inducing hypothalamic secretion of OX-A.

In this experiment, we observed that the activation of NF-κB and its subsequent activation of NLRP3 inflammatory bodies and the release of inflammatory cytokines contribute to the pathophysiological mechanism of TBI. Importantly, we found that LIFUS promoted hypothalamic OX-A secretion and inhibited NF-κB/NLRP3 inflammasome signaling pathway through OX-A. To summarize, LIFUS may alleviate brain injury by inhibiting the inflammatory response after TBI through the OX-A /NF-κB/NLRP3 signaling pathway.

However, this study had some limitations. First, we only discussed the neuroprotective effect of LIFUS in the early stage after brain injury; therefore, it is necessary to study the long-term prognosis effect of LIFUS on brain injury. Second, the selection of ultrasound parameters in this study only referred to previous literatures. In the next step, different ultrasound parameters should be studied and compared to determine the optimal parameter for neuroprotection after TBI. Third, multiple pathological processes can be triggered after TBI, but we only studied the inflammatory response after TBI; therefore, it is uncertain whether LIFUS can achieve neuroprotective functions through other routes. Fourth, in terms of the inflammatory response after TBI, various signaling pathways are involved in the inflammatory response. We only identified the OX-A/NF-κB/NLRP3 pathway, but did not exclude the involvement of other pathways.

## CONCLUSIONS

Our results suggest that the stimulation of the hypothalamus by LIFUS can significantly improve neurological function and reduce brain tissue damage after TBI in rats, and the mechanism may be that LIFUS inhibits the inflammatory response after TBI through the OX-A/NF-κB/NLRP3 signaling pathway. Therefore, LIFUS has the potential and may be effective in treating of patients with TBI.
